# Triethyl­ammonium hydrogen chloranilate

**DOI:** 10.1107/S1600536810047744

**Published:** 2010-11-20

**Authors:** Kazuma Gotoh, Shinpei Maruyama, Hiroyuki Ishida

**Affiliations:** a Department of Chemistry, Faculty of Science, Okayama University, Okayama 700-8530, Japan

## Abstract

In the crystal structure of the title compound (systematic name: triethyl­ammonium 2,5-dichloro-4-hy­droxy-3,6-dioxo­cyclo­hexa-1,4-dien-1-olate), C_6_H_16_N^+^·C_6_HCl_2_O_4_^−^, two hydrogen chloranilate anions are connected by a pair of bifurcated O—H⋯O hydrogen bonds into a dimeric unit. The triethyl­ammonium cations are linked on both sides of the dimer *via* bifurcated N—H⋯O hydrogen bonds into a centrosymmetric 2:2 aggregate. The 2:2 aggregates are further linked by inter­molecular C—H⋯O hydrogen bonds.

## Related literature

For related structures, see, for example: Gotoh *et al.* (2008 ▶, 2009 ▶); Gotoh & Ishida (2009 ▶); Yang (2007 ▶). For details of the double π system of chloranilic acid, see: Andersen (1967 ▶); Benchekroun & Savariault (1995 ▶).
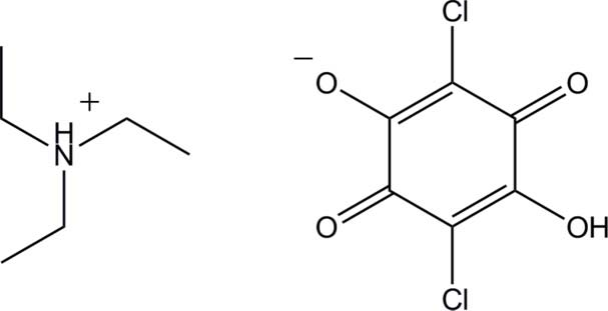


## Experimental

### 

#### Crystal data

C_6_H_16_N^+^·C_6_HCl_2_O_4_^−^
*M* *_r_* = 310.18
Triclinic, 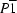

*a* = 7.6404 (5) Å
*b* = 9.5352 (3) Å
*c* = 11.2976 (5) Å
α = 99.9621 (15)°
β = 108.732 (3)°
γ = 106.536 (3)°
*V* = 714.84 (6) Å^3^
*Z* = 2
Mo *K*α radiation
μ = 0.46 mm^−1^
*T* = 180 K
0.42 × 0.35 × 0.25 mm

#### Data collection

Rigaku R-AXIS RAPID II diffractometer
Absorption correction: numerical (*NUMABS*; Higashi, 1999 ▶) *T*_min_ = 0.829, *T*_max_ = 0.891
14757 measured reflections
4176 independent reflections
3631 reflections with *I* > 2σ(*I*)
*R*_int_ = 0.034

#### Refinement

*R*[*F* ^2^ > 2σ(*F* ^2^)] = 0.031
*wR*(*F* ^2^) = 0.092
*S* = 1.07
4176 reflections
180 parameters
H atoms treated by a mixture of independent and constrained refinement
Δρ_max_ = 0.59 e Å^−3^
Δρ_min_ = −0.36 e Å^−3^

### 

Data collection: *PROCESS-AUTO* (Rigaku/MSC, 2004 ▶); cell refinement: *PROCESS-AUTO*; data reduction: *CrystalStructure* (Rigaku/MSC, 2004 ▶); program(s) used to solve structure: *SHELXS97* (Sheldrick, 2008 ▶); program(s) used to refine structure: *SHELXL97* (Sheldrick, 2008 ▶); molecular graphics: *ORTEP-3* (Farrugia, 1997) ▶; software used to prepare material for publication: *CrystalStructure* and *PLATON* (Spek, 2009 ▶).

## Supplementary Material

Crystal structure: contains datablocks global, I. DOI: 10.1107/S1600536810047744/hg2739sup1.cif

Structure factors: contains datablocks I. DOI: 10.1107/S1600536810047744/hg2739Isup2.hkl

Additional supplementary materials:  crystallographic information; 3D view; checkCIF report

## Figures and Tables

**Table 1:** Hydrogen-bond geometry (Å, °)

*D*—H⋯*A*	*D*—H	H⋯*A*	*D*⋯*A*	*D*—H⋯*A*
N1—H1⋯O1	0.847 (18)	2.411 (15)	2.9805 (12)	125.1 (13)
N1—H1⋯O4	0.847 (18)	2.069 (18)	2.8833 (12)	161.1 (14)
O2—H2⋯O3	0.765 (19)	2.147 (19)	2.6331 (11)	121.9 (17)
O2—H2⋯O3^i^	0.765 (19)	2.082 (19)	2.7089 (12)	139.4 (19)
C7—H7*B*⋯O2^ii^	0.99	2.47	3.2859 (15)	140
C8—H8*A*⋯O4^iii^	0.98	2.47	3.3977 (14)	158

Symmetry codes: (i) 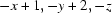; (ii) 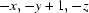; (iii) 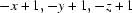.

**Fig. 1.**
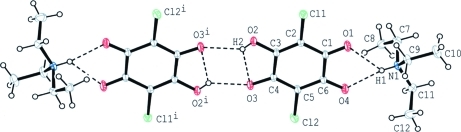
The molecular structure of the title compound, with the atom-labeling. Displacement ellipsoids of non-H atoms are drawn at the 35% probability level. The dashed lines indicate O—H···O and N—H···O hydrogen bonds. [Symmetry code: (i) -x + 1, -y + 2, -z].

**Fig. 2.**
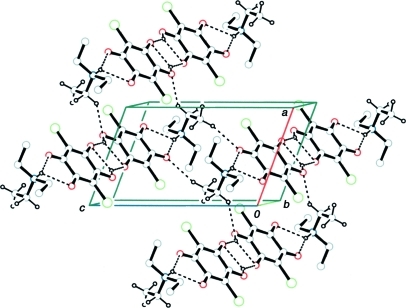
A partial packing diagram of the title compound. The dashed lines indicate O—H···O, N—H···O and C—H···O hydrogen bonds. H atoms of the ethyl groups not involved in the C—H···O hydrogen bonds have been omitted.

## supplementary crystallographic information

### Comment

The title compound, (I), was prepared in order to extend our study on *D*—H···*A* hydrogen bonding (*D* = N, O, or C; *A* = N, O or Cl) in amine–chloranilic acid systems (Gotoh *et al.*, 2008,2009; Gotoh & Ishida, 2009). The crystal structure of bis(hexamethylenetetraminium) chloranilate tetrahydrate has been reported for the tertiary amine–chloranilic acid 2:1 system (Yang, 2007).

In the crystal structure of the title compound, an acid-base interaction involving proton transfer is observed between chloranilic acid and triethylamine, and two hydrogen chloranilate anions and two triethylammnoium cations are linked by bifurcated O—H···O and N—H···O hydrogen bonds (Table 1) to afford a centrosymmetric 2:2 aggregate (Fig. 1). The anion shows a characteristic structure of the double π system (Andersen, 1967; Benchekroun & Savariault, 1995) with two long C1—C6 [1.5442 (13) Å] and C3—C4 [1.5063 (13) Å] bonds. The O3—C4 and O4—C6 bonds [1.2529 (11) and 1.2510 (11) Å, respectively] in one π system are almost same and comparable to the O—C bonds in the dianion of bis(hexamethylenetetraminium) chloranilate tetrahydrate (Yang, 2007). On the other hand, the O1—C1 and O2—C3 bonds [1.2199 (12) and 1.3324 (11) Å, respectively] in the other π system correspond to double and single bonds, respectively. The 2:2 aggregates are further linked by intermolecular C—H···O hydrogen bonds, forming a three-dimensional network (Fig. 2).

### Experimental

Single crystals were obtained by slow evaporation from an acetonitrile solution (25 ml) of chloranilic acid (97 mg) and triethylamine (42 mg) at room temperature.

### Refinement

C-bound H atoms were positioned geometrically (C—H = 0.98 or 0.99 Å) and refined as riding, allowing for free rotation of the methyl group. *U*_iso_(H) values were set at 1.2*U*_eq_(C) or 1.5*U*_eq_(methyl C). The O– and N-bound H atoms were found in a difference Fourier map and refined isotropically. The refined O—H and N—H distances are 0.765 (19) and 0.847 (18) Å, respectively.

### Crystal data

**Table:**

C_6_H_16_N^+^·C_6_HCl_2_O_4_^−^	*Z* = 2
*M**_r_* = 310.18	*F*(000) = 324.00
Triclinic, *P*1	*D*_x_ = 1.441 Mg m^−^^3^
Hall symbol: -P 1	Mo *K*α radiation, λ = 0.71075 Å
*a* = 7.6404 (5) Å	Cell parameters from 12766 reflections
*b* = 9.5352 (3) Å	θ = 3.0–30.1°
*c* = 11.2976 (5) Å	µ = 0.46 mm^−^^1^
α = 99.9621 (15)°	*T* = 180 K
β = 108.732 (3)°	Block, brown
γ = 106.536 (3)°	0.42 × 0.35 × 0.25 mm
*V* = 714.84 (6) Å^3^	

### Data collection

**Table:**

Rigaku R-AXIS RAPID II diffractometer	3631 reflections with *I* > 2σ(*I*)
Detector resolution: 10.00 pixels mm^-1^	*R*_int_ = 0.034
ω scans	θ_max_ = 30.0°
Absorption correction: numerical (*NUMABS*; Higashi, 1999)	*h* = −10→10
*T*_min_ = 0.829, *T*_max_ = 0.891	*k* = −13→13
14757 measured reflections	*l* = −15→15
4176 independent reflections	

### Refinement

**Table:**

Refinement on *F*^2^	Primary atom site location: structure-invariant direct methods
Least-squares matrix: full	Secondary atom site location: difference Fourier map
*R*[*F*^2^ > 2σ(*F*^2^)] = 0.031	Hydrogen site location: inferred from neighbouring sites
*wR*(*F*^2^) = 0.092	H atoms treated by a mixture of independent and constrained refinement
*S* = 1.07	*w* = 1/[σ^2^(*F*_o_^2^) + (0.0543*P*)^2^ + 0.1133*P*] where *P* = (*F*_o_^2^ + 2*F*_c_^2^)/3
4176 reflections	(Δ/σ)_max_ = 0.001
180 parameters	Δρ_max_ = 0.59 e Å^−^^3^
0 restraints	Δρ_min_ = −0.35 e Å^−^^3^

### Special details

**Table:**

Geometry. All e.s.d.'s (except the e.s.d. in the dihedral angle between two l.s. planes) are estimated using the full covariance matrix. The cell e.s.d.'s are taken into account individually in the estimation of e.s.d.'s in distances, angles and torsion angles; correlations between e.s.d.'s in cell parameters are only used when they are defined by crystal symmetry. An approximate (isotropic) treatment of cell e.s.d.'s is used for estimating e.s.d.'s involving l.s. planes.
Refinement. Refinement of *F*^2^ against ALL reflections. The weighted *R*-factor *wR* and goodness of fit *S* are based on *F*^2^, conventional *R*-factors *R* are based on *F*, with *F* set to zero for negative *F*^2^. The threshold expression of *F*^2^ > σ(*F*^2^) is used only for calculating *R*-factors(gt) *etc*. and is not relevant to the choice of reflections for refinement. *R*-factors based on *F*^2^ are statistically about twice as large as those based on *F*, and *R*- factors based on ALL data will be even larger.

### Fractional atomic coordinates and isotropic or equivalent isotropic displacement parameters (Å^2^)

**Table:**

	*x*	*y*	*z*	*U*_iso_*/*U*_eq_	
Cl1	0.05941 (4)	0.44199 (3)	−0.17017 (2)	0.03282 (8)	
Cl2	0.79265 (4)	0.85112 (3)	0.35611 (2)	0.03113 (8)	
O1	0.23295 (13)	0.35023 (9)	0.06660 (8)	0.03546 (18)	
O2	0.29888 (11)	0.77018 (9)	−0.10218 (8)	0.02849 (16)	
O3	0.60830 (11)	0.93820 (8)	0.11497 (7)	0.02933 (16)	
O4	0.53520 (12)	0.51893 (9)	0.29193 (8)	0.03090 (17)	
N1	0.31226 (12)	0.21786 (9)	0.28996 (8)	0.02410 (16)	
C1	0.31746 (14)	0.48352 (11)	0.07566 (9)	0.02382 (18)	
C2	0.25857 (14)	0.55307 (11)	−0.02828 (9)	0.02340 (18)	
C3	0.35511 (14)	0.70234 (11)	−0.01071 (9)	0.02249 (18)	
C4	0.53096 (14)	0.80246 (10)	0.11283 (9)	0.02232 (17)	
C5	0.59095 (14)	0.73644 (11)	0.21385 (9)	0.02326 (18)	
C6	0.49384 (14)	0.58366 (11)	0.20508 (9)	0.02325 (18)	
C7	0.12245 (15)	0.22545 (12)	0.29816 (11)	0.0307 (2)	
H7A	0.0632	0.1399	0.3289	0.037*	
H7B	0.0275	0.2125	0.2098	0.037*	
C8	0.15318 (17)	0.37395 (13)	0.38885 (11)	0.0323 (2)	
H8A	0.2209	0.3765	0.4795	0.048*	
H8B	0.0242	0.3827	0.3768	0.048*	
H8C	0.2344	0.4594	0.3693	0.048*	
C9	0.27183 (18)	0.08580 (12)	0.17789 (11)	0.0320 (2)	
H9A	0.3979	0.0937	0.1676	0.038*	
H9B	0.1802	0.0938	0.0967	0.038*	
C10	0.1828 (2)	−0.06935 (13)	0.19402 (15)	0.0435 (3)	
H10A	0.2787	−0.0831	0.2686	0.065*	
H10B	0.1502	−0.1493	0.1145	0.065*	
H10C	0.0619	−0.0762	0.2094	0.065*	
C11	0.45590 (16)	0.22080 (13)	0.41806 (11)	0.0313 (2)	
H11A	0.3995	0.1271	0.4406	0.038*	
H11B	0.4747	0.3098	0.4872	0.038*	
C12	0.65531 (19)	0.23066 (17)	0.41462 (15)	0.0461 (3)	
H12A	0.6413	0.1348	0.3574	0.069*	
H12B	0.7501	0.2480	0.5031	0.069*	
H12C	0.7037	0.3156	0.3811	0.069*	
H1	0.365 (2)	0.2974 (19)	0.2713 (14)	0.037 (4)*	
H2	0.364 (3)	0.855 (2)	−0.0717 (17)	0.055 (5)*	

### Atomic displacement parameters (Å^2^)

**Table:**

	*U*^11^	*U*^22^	*U*^33^	*U*^12^	*U*^13^	*U*^23^
Cl1	0.03108 (13)	0.02407 (13)	0.03098 (14)	0.00336 (10)	0.00192 (10)	0.00952 (9)
Cl2	0.03405 (14)	0.02543 (13)	0.02406 (13)	0.00126 (10)	0.00727 (10)	0.00770 (9)
O1	0.0406 (4)	0.0198 (4)	0.0355 (4)	0.0021 (3)	0.0070 (3)	0.0134 (3)
O2	0.0304 (4)	0.0204 (4)	0.0303 (4)	0.0052 (3)	0.0067 (3)	0.0140 (3)
O3	0.0342 (4)	0.0184 (3)	0.0333 (4)	0.0056 (3)	0.0114 (3)	0.0128 (3)
O4	0.0353 (4)	0.0252 (4)	0.0283 (4)	0.0050 (3)	0.0091 (3)	0.0153 (3)
N1	0.0257 (4)	0.0190 (4)	0.0277 (4)	0.0046 (3)	0.0123 (3)	0.0098 (3)
C1	0.0266 (4)	0.0192 (4)	0.0267 (4)	0.0069 (3)	0.0112 (3)	0.0101 (3)
C2	0.0235 (4)	0.0195 (4)	0.0255 (4)	0.0059 (3)	0.0079 (3)	0.0088 (3)
C3	0.0244 (4)	0.0203 (4)	0.0257 (4)	0.0082 (3)	0.0113 (3)	0.0109 (3)
C4	0.0252 (4)	0.0183 (4)	0.0262 (4)	0.0076 (3)	0.0124 (3)	0.0092 (3)
C5	0.0260 (4)	0.0193 (4)	0.0230 (4)	0.0051 (3)	0.0094 (3)	0.0083 (3)
C6	0.0261 (4)	0.0206 (4)	0.0249 (4)	0.0069 (3)	0.0117 (3)	0.0103 (3)
C7	0.0232 (4)	0.0292 (5)	0.0376 (5)	0.0063 (4)	0.0120 (4)	0.0104 (4)
C8	0.0319 (5)	0.0344 (6)	0.0357 (5)	0.0164 (4)	0.0144 (4)	0.0131 (4)
C9	0.0415 (6)	0.0214 (5)	0.0327 (5)	0.0066 (4)	0.0187 (4)	0.0070 (4)
C10	0.0536 (7)	0.0218 (5)	0.0571 (8)	0.0075 (5)	0.0306 (6)	0.0101 (5)
C11	0.0301 (5)	0.0330 (5)	0.0312 (5)	0.0120 (4)	0.0103 (4)	0.0127 (4)
C12	0.0337 (6)	0.0490 (8)	0.0561 (8)	0.0214 (5)	0.0146 (5)	0.0117 (6)

### Geometric parameters (Å, °)

**Table:**

Cl1—C2	1.7133 (10)	C7—H7A	0.9900
Cl2—C5	1.7307 (10)	C7—H7B	0.9900
O1—C1	1.2199 (12)	C8—H8A	0.9800
O2—C3	1.3324 (11)	C8—H8B	0.9800
O2—H2	0.766 (18)	C8—H8C	0.9800
O3—C4	1.2529 (11)	C9—C10	1.5115 (16)
O4—C6	1.2510 (11)	C9—H9A	0.9900
N1—C11	1.4993 (13)	C9—H9B	0.9900
N1—C9	1.5033 (13)	C10—H10A	0.9800
N1—C7	1.5036 (13)	C10—H10B	0.9800
N1—H1	0.847 (16)	C10—H10C	0.9800
C1—C2	1.4564 (13)	C11—C12	1.5130 (16)
C1—C6	1.5442 (13)	C11—H11A	0.9900
C2—C3	1.3490 (13)	C11—H11B	0.9900
C3—C4	1.5063 (13)	C12—H12A	0.9800
C4—C5	1.4092 (13)	C12—H12B	0.9800
C5—C6	1.4036 (13)	C12—H12C	0.9800
C7—C8	1.5047 (16)		
			
C3—O2—H2	106.0 (13)	C7—C8—H8A	109.5
C11—N1—C9	113.52 (8)	C7—C8—H8B	109.5
C11—N1—C7	111.98 (8)	H8A—C8—H8B	109.5
C9—N1—C7	111.23 (8)	C7—C8—H8C	109.5
C11—N1—H1	107.6 (10)	H8A—C8—H8C	109.5
C9—N1—H1	105.4 (10)	H8B—C8—H8C	109.5
C7—N1—H1	106.5 (10)	N1—C9—C10	114.04 (9)
O1—C1—C2	123.39 (9)	N1—C9—H9A	108.7
O1—C1—C6	118.01 (8)	C10—C9—H9A	108.7
C2—C1—C6	118.60 (8)	N1—C9—H9B	108.7
C3—C2—C1	120.43 (9)	C10—C9—H9B	108.7
C3—C2—Cl1	121.32 (7)	H9A—C9—H9B	107.6
C1—C2—Cl1	118.21 (7)	C9—C10—H10A	109.5
O2—C3—C2	121.58 (9)	C9—C10—H10B	109.5
O2—C3—C4	115.99 (8)	H10A—C10—H10B	109.5
C2—C3—C4	122.42 (8)	C9—C10—H10C	109.5
O3—C4—C5	126.59 (9)	H10A—C10—H10C	109.5
O3—C4—C3	115.62 (8)	H10B—C10—H10C	109.5
C5—C4—C3	117.79 (8)	N1—C11—C12	112.26 (10)
C6—C5—C4	123.24 (9)	N1—C11—H11A	109.2
C6—C5—Cl2	118.80 (7)	C12—C11—H11A	109.2
C4—C5—Cl2	117.95 (7)	N1—C11—H11B	109.2
O4—C6—C5	126.67 (9)	C12—C11—H11B	109.2
O4—C6—C1	115.87 (8)	H11A—C11—H11B	107.9
C5—C6—C1	117.46 (8)	C11—C12—H12A	109.5
N1—C7—C8	112.65 (8)	C11—C12—H12B	109.5
N1—C7—H7A	109.1	H12A—C12—H12B	109.5
C8—C7—H7A	109.1	C11—C12—H12C	109.5
N1—C7—H7B	109.1	H12A—C12—H12C	109.5
C8—C7—H7B	109.1	H12B—C12—H12C	109.5
H7A—C7—H7B	107.8		
			
O1—C1—C2—C3	178.24 (10)	C3—C4—C5—Cl2	−179.88 (7)
C6—C1—C2—C3	−0.79 (14)	C4—C5—C6—O4	−177.80 (10)
O1—C1—C2—Cl1	0.38 (14)	Cl2—C5—C6—O4	1.38 (15)
C6—C1—C2—Cl1	−178.65 (7)	C4—C5—C6—C1	2.18 (14)
C1—C2—C3—O2	−177.16 (9)	Cl2—C5—C6—C1	−178.64 (6)
Cl1—C2—C3—O2	0.63 (14)	O1—C1—C6—O4	−0.58 (14)
C1—C2—C3—C4	2.41 (15)	C2—C1—C6—O4	178.50 (9)
Cl1—C2—C3—C4	−179.80 (7)	O1—C1—C6—C5	179.44 (9)
O2—C3—C4—O3	−1.57 (12)	C2—C1—C6—C5	−1.48 (13)
C2—C3—C4—O3	178.84 (9)	C11—N1—C7—C8	64.50 (11)
O2—C3—C4—C5	177.84 (8)	C9—N1—C7—C8	−167.30 (9)
C2—C3—C4—C5	−1.75 (14)	C11—N1—C9—C10	60.54 (13)
O3—C4—C5—C6	178.64 (9)	C7—N1—C9—C10	−66.83 (13)
C3—C4—C5—C6	−0.70 (14)	C9—N1—C11—C12	59.27 (12)
O3—C4—C5—Cl2	−0.55 (14)	C7—N1—C11—C12	−173.75 (9)

### Hydrogen-bond geometry (Å, °)

**Table:**

*D*—H···*A*	*D*—H	H···*A*	*D*···*A*	*D*—H···*A*
N1—H1···O1	0.847 (18)	2.411 (15)	2.9805 (12)	125.1 (13)
N1—H1···O4	0.847 (18)	2.069 (18)	2.8833 (12)	161.1 (14)
O2—H2···O3	0.765 (19)	2.147 (19)	2.6331 (11)	121.9 (17)
O2—H2···O3^i^	0.765 (19)	2.082 (19)	2.7089 (12)	139.4 (19)
C7—H7B···O2^ii^	0.99	2.47	3.2859 (15)	140
C8—H8A···O4^iii^	0.98	2.47	3.3977 (14)	158

Symmetry codes: (i) −*x*+1, −*y*+2, −*z*; (ii) −*x*, −*y*+1, −*z*; (iii) −*x*+1, −*y*+1, −*z*+1.

## References

[bb1] Andersen, E. K. (1967). *Acta Cryst.* **22**, 196–201.

[bb2] Benchekroun, R. & Savariault, J.-M. (1995). *Acta Cryst.* C**51**, 186–188.

[bb3] Farrugia, L. J. (1997). *J. Appl. Cryst.* **30**, 565.

[bb4] Gotoh, K., Asaji, T. & Ishida, H. (2008). *Acta Cryst.* C**64**, o550–o553. 10.1107/S0108270108029028 18838773

[bb5] Gotoh, K. & Ishida, H. (2009). *Acta Cryst.* E**65**, o2467. 10.1107/S1600536809036605 PMC2970440 21577921

[bb6] Gotoh, K., Nagoshi, H. & Ishida, H. (2009). *Acta Cryst.* C**65**, o273–o277. 10.1107/S010827010901525X 19498235

[bb7] Higashi, T. (1999). *NUMABS* Rigaku Corporation, Tokyo, Japan.

[bb8] Rigaku/MSC. (2004). *PROCESS-AUTO* and *CrystalStructure* Rigaku/MSC Inc., The Woodlands, Texas, USA.

[bb9] Sheldrick, G. M. (2008). *Acta Cryst.* A**64**, 112–122. 10.1107/S0108767307043930 18156677

[bb10] Spek, A. L. (2009). *Acta Cryst.* D**65**, 148–155. 10.1107/S090744490804362X PMC2631630 19171970

[bb11] Yang, D.-J. (2007). *Acta Cryst.* E**63**, o2600.

